# Quantification of the effects of climatic conditions on French hospital admissions and deaths induced by SARS-CoV-2

**DOI:** 10.1038/s41598-021-01392-2

**Published:** 2021-11-08

**Authors:** Hippolyte d’Albis, Dramane Coulibaly, Alix Roumagnac, Eurico de Carvalho Filho, Raphaël Bertrand

**Affiliations:** 1 Paris School of Economics, CNRS, 48 Boulevard Jourdan, 75014 Paris, France; 2 Univ Lyon, Université Lumière Lyon 2, GATE, 93, Chemin des Mouilles, B.P. 167, 69131 Ecully Cedex, France; 3 PREDICT Services, 20 Rue Didier Daurat, 34170 Castelnau-le-Lez, France

**Keywords:** Statistics, Infectious diseases

## Abstract

An estimation of the impact of climatic conditions—measured with an index that combines temperature and humidity, the IPTCC—on the hospitalizations and deaths attributed to SARS-CoV-2 is proposed. The present paper uses weekly data from 54 French administrative regions between March 23, 2020 and January 10, 2021. Firstly, a Granger causal analysis is developed and reveals that past values of the IPTCC contain information that allow for a better prediction of hospitalizations or deaths than that obtained without the IPTCC. Finally, a vector autoregressive model is estimated to evaluate the dynamic response of hospitalizations and deaths after an increase in the IPTCC. It is estimated that a 10-point increase in the IPTCC causes hospitalizations to rise by 2.9% (90% CI 0.7–5.0) one week after the increase, and by 4.1% (90% CI 2.1–6.4) and 4.4% (90% CI 2.5–6.3) in the two following weeks. Over ten weeks, the cumulative effect is estimated to reach 20.1%. Two weeks after the increase in the IPTCC, deaths are estimated to rise by 3.7% (90% CI 1.6–5.8). The cumulative effect from the second to the tenth weeks reaches 15.8%. The results are robust to the inclusion of air pollution indicators.

## Introduction

SARS-CoV-2 appeared in China in 2019 and has produced a global pandemic—the COVID-19 pandemics—as of March 2020^1^. To cope with the disease, unprecedented mobility reduction measures such as lockdowns or curfews were implemented in many countries around the world, causing a major financial impact. These measures were justified by the fact that population mobility is a key factor for the virus circulation^2^, together with population density, associated with a higher likelihood of infectious contacts between people^3^.

Among other causes, several studies have shown the link between the spread of respiratory viruses and climatic conditions^4,5^. This is the case of the influenza virus, for which the role of absolute humidity on transmission and seasonality has been demonstrated^6^. There are several types of transmission for respiratory viruses, but airborne transmission by aerosols (small particles) is more likely to be impacted by meteorological conditions. However, the mechanisms involved in these processes are still poorly understood, which calls for further investigations^7^.

Concerning the SARS-CoV-2 outbreak, a pioneering study^8^ conducted at the beginning of the pandemic warned that 90% of infections occurred in areas with temperatures between 3 and 17 °C, with an absolute humidity between 4 and 9 g/m^3^. Other studies from various countries have linked temperature or humidity to COVID-19. Most notably, it has been shown that for Northern hemisphere countries with a mean regional temperature below 10 °C, a variation of 1 g/m^3^ of the average absolute humidity can be associated with a variation of 0.15-unit in the basic reproduction number—the R0—and an increase of 1 °C can be associated with a 0.16-unit lower R0^9^.

In the present paper, a statistical investigation is proposed using a recently developed indicator created by PREDICT Services, known as the IPTCC, an acronym in French for PREDICT’s Index for COVID-climate transmissivity^10^. This index was notably used by the Pasteur Institute to refine their models and to integrate an environmental factor that aims at better explaining the evolution of epidemiological indexes. Furthermore, the integration of the IPTCC into statistical models (such as the Multiple Linear Regression Model and the Generalized Additive Model) corrected the error between the forecasts and the observations of hospital admissions by 22% and 13% respectively^11^.

The virus circulation was spatially and temporally irregular across mainland France in 2020. A first wave affected the country between March and April 2020, particularly the Paris region and the Northeast half of the country. The number of contaminations dropped sharply in late spring and remained considerably low in summer. Then, a second wave homogeneously impacted the country between October and November 2020. Since December 2020, the situation has reached a plateau with a high number of daily cases.

From the meteorological perspective, France is exposed to several climate dynamics. The western shoreline has an oceanic climate characterized by mild temperatures and significant rainfall throughout the year. The northeast of the country has a semi-continental climate with hot summers and cold winters. The Mediterranean climate is characterized by mild winters, warm summers, and less rainfall, which is irregular.

We take advantage of this geographical diversity to conduct a causal analysis of climatic conditions on the hospital admissions and deaths induced by SARS-CoV-2. Our approach is based on the estimation of vector autoregressive (VAR) models, following an established practice in economics since^12^, where they are used to quantify an economy's response to an exogenous structural shock; they are also used in the life sciences (see e.g.^13,14^), and were found to be useful in particular to analyze the dynamics of SARS-CoV-2 (^15,16^). VAR models can be easily used to quantify the effects of climatic conditions on epidemiological variables, as the former are clearly exogenous to the latter.

Our analysis is conducted at the regional level to exploit the heterogeneity (both in terms of climatic conditions and prevalence of the virus) across regions and to obtain an estimation that is not biased by sanitary measures. Over the period considered, mobility restrictions implemented by the government were indeed generally the same in all regions of mainland France. Specifically, a strict national lockdown was imposed from March 17, 2020 to May 11, 2020 and a mild one (with schools that remained open) from October 30, 2020 to December 15, 2020.

## Methods

### Data

We considered 54 NUTS3 administrative regions (named *départements* in French) over 42 weeks (from March 23, 2020 to January 10, 2021). We did not consider all existing regions, but only the 54 for which the IPTCC can be computed. In order to evaluate the consequences of the climatic conditions on the pandemic, we used the weekly numbers of hospital admissions and deaths due to SARS-CoV-2 for the same 54 administrative regions considered. The data are official and publicly available at www.data.gouv.fr. The hospitalizations and deaths due to SARS-CoV-2 were consistently measured over the period and are thus more reliable than the series that report the number of infections, as the latter highly depend on the availability of tests and on the population’s willingness to get tested. Daily data are available since March 19, 2020, but we chose to use the weekly frequency in order to avoid a seasonal effect induced by lower reporting during weekends.

The meteorological data came from the 63 Météo-France stations. These stations are homogeneously distributed over mainland France and they were chosen in order to better represent the diversity of the country’s climate. The dataset provides a daily average for air temperature (measured in °C) and relative humidity (measured in %); these values were calculated as an average of the daily maximum and minimum values of temperature and relative humidity. With them, it is easy to compute the daily absolute humidity for each station, measured in g/m^3^, using the Clausius-Clapeyron equation^8^.

In order to analyze the potential relation between climate conditions and virus transmission, the IPTCC was created to characterize the potential for virus transmission according to climatic conditions. Following^10^, the IPTCC is a function of absolute humidity (AH), relative humidity (RH), and temperature (T), and the formula can be written as follows:

$$IPTCC = 100* e^{{ - \frac{1}{2}\left[ {\frac{{\left( {T - 7.5} \right)^{2} }}{196} + \frac{{\left( {RH - 75} \right)^{2} }}{625} + \frac{{\left( {AH - 6} \right)^{2} }}{2.89}} \right]}} .$$

The IPTCC is thus maximal when the temperature reaches 7.5 °C and the relative humidity 75%. The IPTCC is available on a daily basis since January 1, 2020 for all meteorological stations. Then, for each of the 54 regions considered, we built a weekly indicator computed as the average of the daily IPTCC. Visualizations of the IPTCC through 2D and 3D representations are provided in the Supplementary Materials (Figs. A1 and A2).

**Figure 1:**
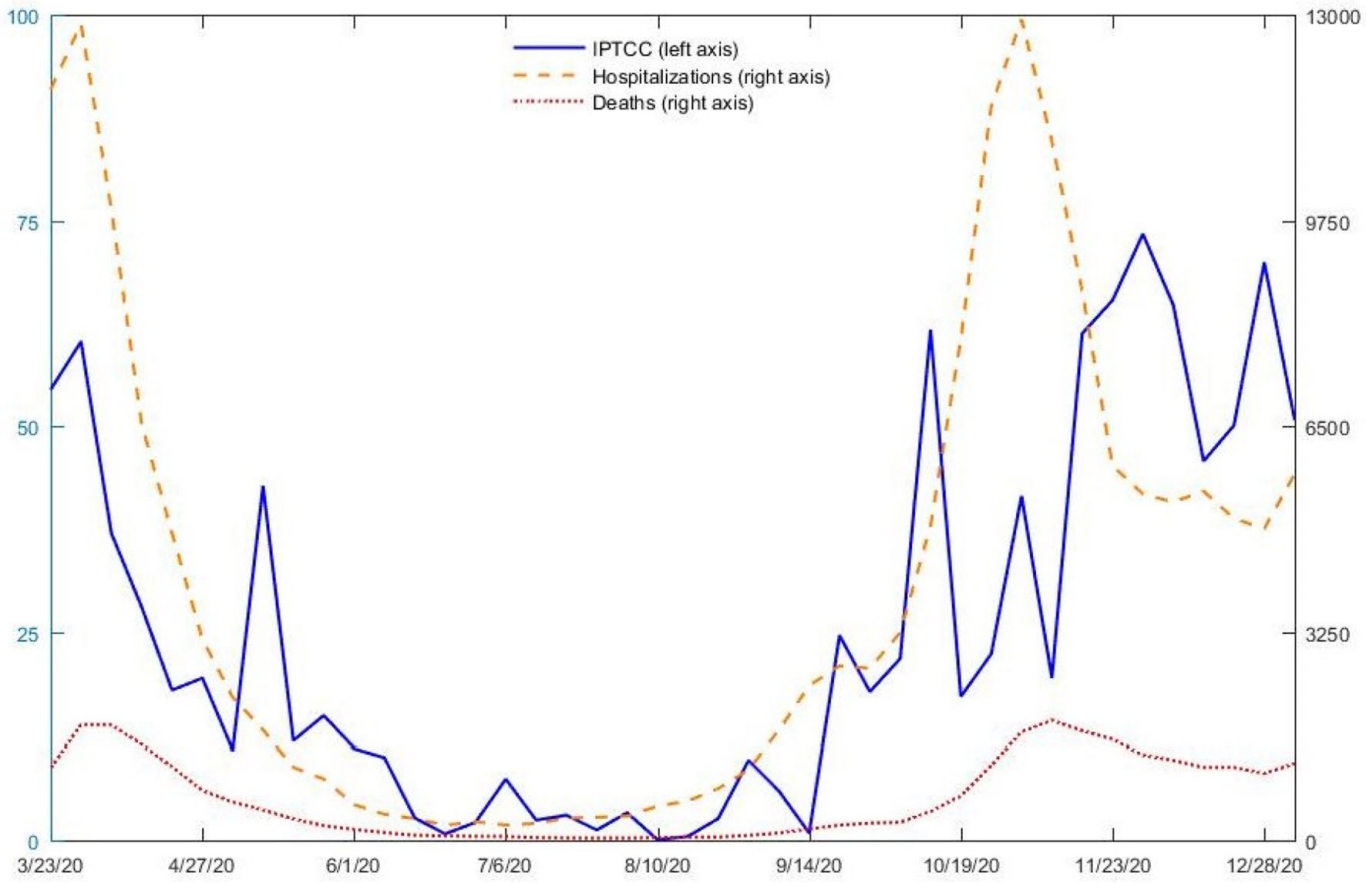
Comparison of the time series of total hospitalizations and deaths and the average IPTCC, March 23, 2020 to January 10, 2021.

As a preliminary step, the evolution of the variables over the period considered can be plotted. The figures for each of the 54 regions are reported in the Supplementary Materials (Fig. A3). To summarize them, Fig. 1 reports weekly hospitalizations and deaths (i.e. the total for the 54 regions) and the weekly IPTCC (the average for the 54 regions). The correlation between the three series is quite remarkable (see Table A2 for the coefficients). Most notably, the peaks in hospitalizations and deaths correspond to periods during which the IPTCC is high on average. Conversely, throughout spring and summer, hospitalizations, deaths, and the IPTCC are low. We also notice that the IPTCC is more volatile than the epidemiological series. Lastly, the evolution of the IPTCC seems to precede those of hospitalizations and deaths, which is probably due to the time between contamination, the incubation period, and a possible worsening of the disease.

To go beyond this first graphical analysis and investigate how climatic conditions affect the spread and severity of SARS-CoV-2, we developed a time series analysis. This was done in two steps. Firstly, we demonstrated a causal relationship between the IPTCC and the hospitalization and deaths, and then we evaluated the responses of the latter to an increase in the IPTCC.

### Granger causality

We firstly explored whether climate conditions Granger-cause hospitalizations and deaths from SARS-CoV-2, i.e. whether the past values of climatic conditions contain information that are helpful to predict hospitalizations or deaths given that past hospitalization and death information are considered.

To test for Granger causality from climate conditions to hospitalizations and deaths, we considered the following panel dynamic model using the weekly data for 54 regions, from March 23, 2020 to January 10, 2021.

1 $$\begin{array}{*{20}c} {y_{it} = \mathop \sum \limits_{s = 1}^{p} \alpha_{s} y_{it - s} + \mathop \sum \limits_{s = 1}^{p} \beta_{s} cl_{it - s} + \mu_{i} + \delta_{i} .t + \eta_{t} + \varepsilon_{it} , i = 1, \ldots N\; and\; t = 1, \ldots ,T} \\ \end{array}$$

where $i$ and $t$ stand respectively for the indices of region and time; $y_{it} \in \left\{ {hosp_{it} ,death_{it} } \right\}$ with $hosp_{it}$ and $death_{it}$ respectively denote the logarithm of 1 plus the number of hospitalizations and deaths; $cl_{it}$ represents the IPTCC index; $\mu_{i}$ are regional fixed effects, $\delta_{i} t$ are regional-specific time (linear) trends, and $\eta_{t}$ is the common national time (week)-specific effect including seasonal effect.

It is worth noting that taking the logarithm of hospitalizations and deaths allows, through regional fixed effects $u_{i}$, to account for regional heterogeneities such as area, population, or density, which are roughly stable over the considered period. Moreover, including the region-specific time trend $\delta_{i} .t$ with the log specification may capture a potential exponential growth of the disease spreading that could be region specific. Finally, using $\eta_{t}$ allows taking into account the national interdependence (including any seasonal effect) of the disease spreading across regions. Preliminary diagnostics (panel unit root tests) rejected the null hypothesis of unit root for the de-trended variables (with a region-specific linear trend). We then considered variables in level or log level while controlling for region heterogeneity (by introducing region-specific effects and region-specific time trends) and cross-region interdependence (by introducing week-specific effects).

Let ${\uptheta } = \left( {\alpha_{1} , \ldots ,\alpha_{p} ,\beta_{1} , \ldots ,\beta_{p} } \right)^{\prime}$ be the vector of parameters across cross-section units to be estimated. Given the sizes of the cross-region dimension $N$ and the time dimension $T$ in the panel data ($N = 54$ and $T = 42$), in order to deal with short-T dynamic panel data bias or the so-called Nickell bias^17^, we used the bias-corrected fixed-effect estimator developed by^18^, which is appropriate when $0 < \lim N/T < \infty$, as is the case here (see^19^). This technique consists in removing the asymptotic bias of least square dummy variable (LSDV) or fixed effect estimator (with region-specific time trends and time-specific effects) of $\uptheta$. The LSDV estimator of $\uptheta$ which is given by the ordinary least square (OLS) regression of $\tilde{y}_{it}$ on $\tilde{y}_{it - 1} , \ldots ,\tilde{y}_{it - p} ,\widetilde{cl}_{it - 1} , \ldots ,\widetilde{cl}_{it - p}$ where $\tilde{x}_{it}$ is a transformation of $x_{it}$ after removing region specific effects and trends and the national average for each week (this transformation corresponds to include $\mu_{i}$, $\delta_{i} \cdot t$, and $\eta_{t}$).

Based on the Bayesian information criterion (BIC) and the Hannan-Quinn information criterion (HQC), we set the lag length $p$ to 3, so that there is no serial correlation in the errors. Taking a lag length higher than 3 does not alter our findings.

In Eq. (1), the null hypothesis of no Granger causality from the climate conditions ($cl_{it}$) to hospitalizations or deaths ($y_{it} = hosp_{it} \;{\text{or}}\; death_{it} )$ is $H_{0} : \beta_{1} = \beta_{2} = \ldots = \beta_{p}$. The null hypothesis of no Granger causality can be expressed as $R{\uptheta } = 0_{p \times 1}$ where $R$ is a known $\left( {p \times 2p} \right)$ matrix with $R = \left[ {0:I_{p} } \right]$. The test statistics, which is a Wald statistics, is given by $W = \hat{\theta }^{\prime}R^{\prime}\left[ {\hat{\sigma }^{2} R\left( {X^{\prime}X} \right)^{ - 1} R^{\prime}} \right]^{ - 1} R\hat{\theta }$ where $X$ is a $\left( {N\left( {T - p} \right) \times 2p} \right)$ matrix of regressors $\tilde{y}_{it - 1} , \ldots ,\tilde{y}_{it - p} ,\widetilde{cl}_{it - 1} , \ldots ,\widetilde{cl}_{it - p}$ in column and $\hat{\sigma }^{2}$ is the estimated variance of residual. Under the null hypothesis $W$ follows a chi-squared distribution of a degree of freedom equal to $p$, i.e. the number of constraints to be tested that corresponds to the lag length.

### The VAR model

To analyze the dynamic responses of the epidemiological variables to a change in the IPTCC, we estimated a panel vector autoregressive (VAR) model where IPTCC ($cl$) is considered exogenous. It can be written as:

2 $$\begin{array}{*{20}c} {Z_{it} = \mathop \sum \limits_{s = 1}^{p} A_{s} Z_{it - s} + \mathop \sum \limits_{s = 0}^{p} b_{s} cl_{it - s} + u_{i} + d_{i} .t + f_{t} + v_{it} , i = 1, \ldots N\; and \;t = 1, \ldots ,T} \\ \end{array}$$

where $Z_{it} = \left( {hosp_{it} ,death_{it} } \right)^{\prime}$ is a 2-dimensional vector of endogenous variables including the logarithm of 1 plus the hospitalizations and deaths, where $A_{s}$ are $\left( {2 \times 2} \right)$ matrices of coefficients associated with $Z_{it}$, $b_{s}$ are $\left( {2 \times 1} \right)$ matrices associated with $cl_{it - s}$, $u_{i} = \left( {u_{i}^{1} , u_{i}^{2} } \right)^{\prime}$ is a vector of regional fixed-effects; $d_{i} .t = \left( {d_{i}^{1} ,d_{i}^{2} } \right)^{\prime}.t$ represent region specific-time (linear) trend, $f_{t} = \left( {f_{t}^{1} ,f_{t}^{2} } \right)$ is a vector of common time (week)-specific effect; $v_{it} = \left( {v_{it}^{1} ,v_{it}^{2} } \right)^{\prime}$ is a 2-dimensional vector of errors satisfying $E\left( {v_{it} } \right) = 0$ and $E\left( {v_{it} v_{is}^{^{\prime}} } \right) =\Omega .1\left\{ {t = s} \right\}$ for all $t$ and $s$.

As mentioned above, preliminary diagnostics (panel unit root tests) rejected the null hypothesis of unit root for the de-trended variables (with a region-specific linear trend). Our VAR then considers variables in level or log level while controlling for region heterogeneity (by introducing region-specific effects and region-specific time trends) and cross-region interdependence (by introducing week-specific effects). Moreover, the model was estimated through the bias-corrected fixed-effect estimator developed by^18^, and the lag length $p$ was set to 3 based on the Bayesian information criterion (BIC) and the Hannan-Quinn information criterion (HQC). Using a lag length higher than 3 does not change our findings.

After having estimated model (2), we computed the responses of the endogenous variables (hospitalizations and deaths) to an (exogenous) increase in the IPTCC, and the response of deaths to an increase in hospitalizations. The responses of hospitalizations and deaths (endogenous variables) to climate conditions (exogenous variable) is part of the multiplier analysis; for this reason, there is no need to identify the structural shocks of the endogenous variables (see^20^, Sect. 10.6). However, to identify the response of deaths to hospitalization, it is necessary to identify the structural shocks $\eta_{it}$ of these two endogenous variables as follows: $\eta_{it} = A_{0} v_{it}$ where $A_{0}$ is $2 \times 2$ matrix such that $E(\eta_{it} \eta_{it}^{^{\prime}} ) = I_{2}$ or $A_{0} A_{0}^{^{\prime}} = \Omega$. We identified $A_{0}$ based on Cholesky decomposition by setting $A_{0}$ as the unique lower-triangular Cholesky factor of $\Omega$. This identification relies on the reasonable assumption that hospitalizations can influence deaths contemporaneously, while deaths can potentially influence hospitalizations only with lags.

## Results

### Causality between the IPTCC and the epidemiological variables

The results of Granger non-causality from climate conditions to hospitalizations and deaths induced by SARS-CoV-2 are reported in Table 1. At the 5% level of significance, we cannot accept the null hypothesis of no Granger causality from climate conditions to either hospitalizations or deaths. In other words, past information on climate conditions is helpful to predict hospitalizations and deaths even when accounting for past hospitalization and death information.

**Table 1:** Granger causality from the IPTCC to hospitalizations and deaths.

Hypothesis	Test statistics	*P*-value
IPTCC does not Granger-cause hospitalizations	22.044	0.000
IPTCC does not Granger-cause deaths	10.815	0.013

The test statistic is a Wald statistic which follows, under the null hypothesis, a chi-squared distribution of 3 (the number of constraints that corresponds to the lag length).

Although the Granger causality analysis conducted above indicates how the past values of the IPTCC are useful to predict hospitalizations and deaths, it does not provide any evaluation of the response of hospitalizations and deaths to a change in the IPTCC. This is done with the VAR model.

### Estimations of the epidemiological responses to a change in the IPTCC

We consider a 10-point increase in the IPTCC, an increase which is understood as relative to the average IPTCC value over the period considered (namely 25.5). One should note that a 10-point increase correspond to rather small changes in temperature and humidity. For instance, the IPTCC moved from 42 to 52 in the Seine-Maritime *département* between April 6 and April 13 of 2020; this change was due to a decrease in temperature from 13 to 11 °C and an increase in relative humidity from 73 to 75%. The largest increase (76.9) was recorded in the Finistère *département* between May 4 and May 11 of 2020 when temperature fall from 16 to 10 °C and relative humidity declined from 83 to 68%. The largest decline (− 66.9) was observed in the Hérault *département* between December 7 and December 14 of 2020 when temperature rose from 6 to 12 °C and the relative humidity from 76 to 91%.

**Figure 2:**
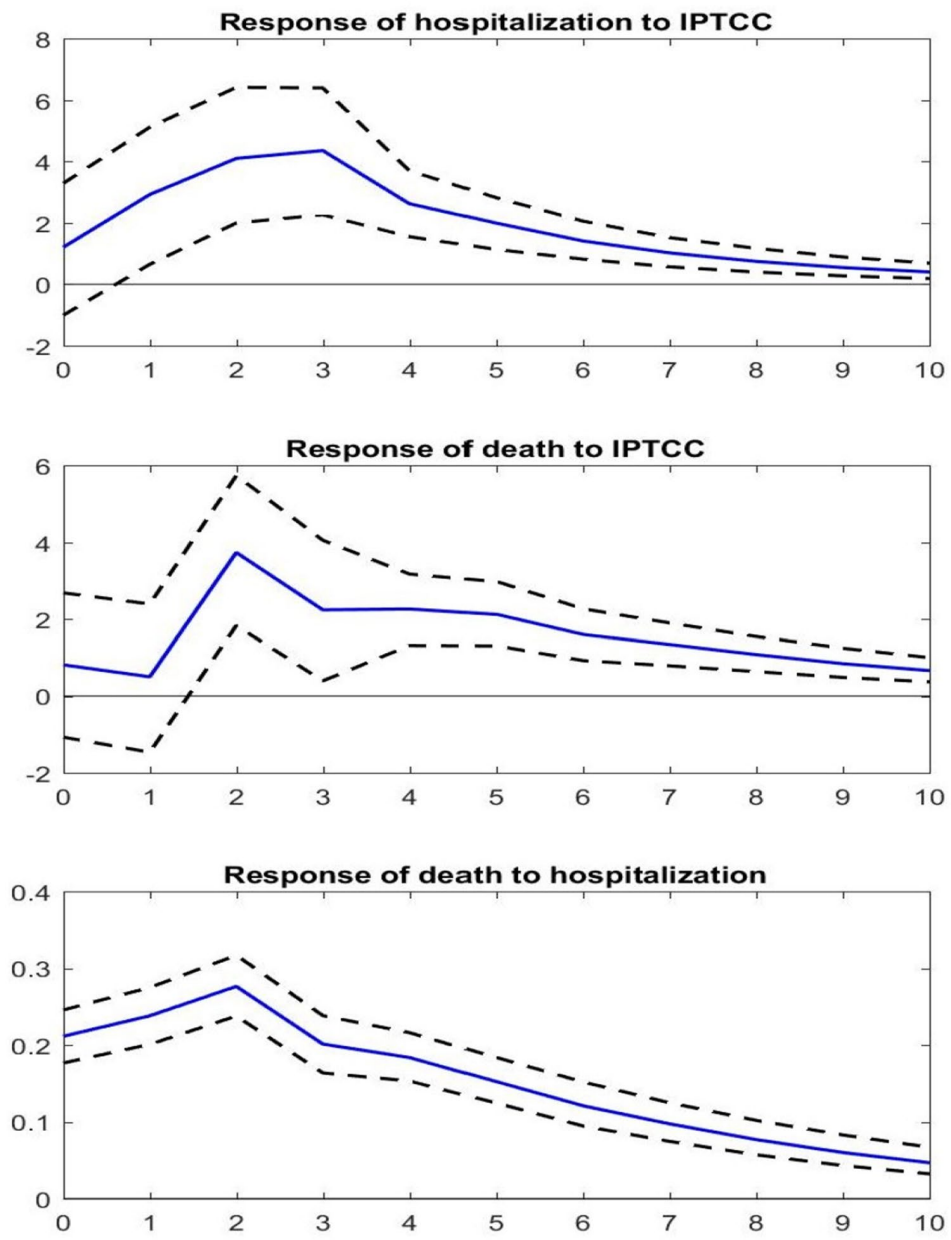
Dynamic responses of hospitalizations and deaths to a shock on the IPTCC. *Note*: The solid line gives the estimated impulse responses. The dashed lines give the 90% confidence intervals generated by Monte Carlo with 5000 repetitions. The size of the increase in the IPTCC is set to a 10-point increase. The size of the increase in hospitalizations is set to a one-percent increase. The responses are the percentage change in the number of hospitalizations and deaths.

The dynamic responses of hospitalizations and deaths to a 10-point increase in the IPTCC are displayed in Fig. 2. The upper panel represents the estimated impact of the IPTCC on hospitalizations. Week 0 is the week when the increase occurs. We observe that the response of hospitalizations is delayed but quite persistent: it is significantly positive from one week after the increase until at least 10 weeks later. In terms of magnitude, we estimate that hospitalizations rise by 2.9% (90% CI 0.7–5.0) one week after the increase in the IPTCC, and by 4.1% (90% CI 2.1–6.4) and 4.4% (90% CI 2.5–6.3) in the two following weeks. Over ten weeks, the cumulative effect is estimated to reach 20.1%. Compared to their average value over the period, i.e. 147 individuals per week and region, this represents 30 individuals. Note that to evaluate e.g. the effect of a 50-point increase that would put the IPTCC over 75, our estimates should simply be multiplied by 5. The cumulative effect on hospitalizations is thus expected to reach 100%.

The middle panel of Fig. 2 represents the estimated impact of the same 10-point increase in the IPTCC on deaths. The impact is also positive but is further delayed, as it becomes positive as of the second week after the increase in the IPTCC. That week, we estimate that the deaths increase by 3.7% (90% CI 1.6–5.8). The cumulative effect from the second to the tenth weeks reaches 15.8%. Compared to their average value over the period, i.e. 25 individuals per week and region, this represents 4 individuals. The link between hospitalizations and deaths is further investigated in the bottom panel of Fig. 2, which represents the response of the number of deaths to a 1% increase in the number of hospitalizations. We observe an immediate response, of magnitude 0.21%, which grows up to 0.28% the second week, and then decreases. These last evaluations are roughly consistent with the existing evaluations (^21^, among others); they are not immediately comparable since we take into account the reaction of hospitalizations to their own change. The existence of persistence in the series explains why our evaluation is slightly higher.

### Sensitivity and robustness analysis

The sensitivity and robustness of our findings were assessed by analyzing the dynamics under alternative proxies for climate conditions and by considering the environmental pollution as a possible interfering variable.

Firstly, it seems that alternative indicators for climatic conditions are less effective than the IPTCC to capture the effect of climate on the epidemiological dynamics. We exemplified this with two alternative indicators. Initially, we used a ‘false’ IPTCC index computed using aberrant target values for temperature and humidity. The ‘false’ IPTCC reaches 100 when the temperature reaches 30 °C and the relative humidity 20%. We obtained that hospitalizations no longer respond significantly to the indicator (see Fig. A4 in the Supplementary Materials). Then, we replaced the IPTCC by a normalized temperature index computed as $T_{n} = 100 \cdot e^{{ - \left( {T - 7.5} \right)}}$, and therefore abstracted from the humidity variables. We obtained that, after three weeks, the response of hospitalizations becomes significant, but the effects are less clear and the magnitude is less important (see Fig. A5 in the Supplementary Materials). This confirms that, although partly correlated, temperature and humidity are both useful when combined in a single indicator to evaluate the effects of climate on epidemiological variables.

Secondly, it is important to investigate whether our results could be biased by some omitted-variables. In particular, environmental pollution, whose effect on respiratory diseases is well established (see e.g.^22^), has been recently shown to be correlated with the prevalence of COVID-19 (^23–25^). We have thus estimated extended versions of our models in order to take into account the evolution of the environmental pollution. More specifically, we have collected weekly averages of the concentration of atmospheric particulate matter (PM_10_ and PM_2.5_), of nitrogen dioxide (NO_2_) and of ozone (O_3_) for a subsample of the regions we considered. We found that, at the 5% level of significance, we still cannot accept the null hypothesis of no Granger causality from climate conditions to either hospitalizations or deaths (Table A3 in the Supplementary Materials). Moreover, the dynamic responses of hospitalizations and deaths to a 10-point increase in the IPTCC are qualitatively similar to those displayed in Fig. 2 (Fig. A6 in the Supplementary Materials). Our results are thus robust to the inclusion of environmental pollution variables in the models. Interestingly, we also obtained that the null of hypothesis of no Granger causality from air pollution to either hospitalizations or deaths cannot be rejected but that, on the short run, the concentration of atmospheric particulate matter has an impact on hospitalizations and deaths.

## Discussion

By testing the statistical impact of the IPTCC on hospital admissions and deaths induced by SARS-CoV-2 in France, this study confirms the potential role of temperature and humidity in the airborne spread of SARS-CoV-2 by highlighting that there is a particular combination of the two variables that creates a favorable ground for the transmission of the disease: when the distance toward a temperature of 7.5 °C and a relative humidity of 75% reduces, hospital admissions and deaths increase. With respect to earlier studies, it provides strong causal evidences and offers a new quantification of the effect of the climate factors on the dynamics of the disease. Most notably, it characterizes the time delay between the climatic conditions and the hospitalizations and deaths and include air pollution indicators as controls.

This study has several limitations, notably it was performed for continental France, and needs to be replicated in other countries in order to ensure that the benchmark combination for which the IPTCC is maximal (temperature at 7.5 °C and relative humidity at 75%) is same. Furthermore, although the model is using regional data, the results are not region specific. They should be interpreted as the response of an average region in France. More data are needed in order to be able to characterize the regional differences. Moreover, the study covers a period that starts in March, 2020 and ends in January, 2021. It thus does not take into account the more recent virus variants nor the vaccination campaign. More generally, it is clear that anthropogenic factors related to the behavior and respect of sanitary recommendations among the citizens, the conditions of hygiene, the access to care, and the quality and resources of the health services have an essential impact on COVID-19 transmission and other indicators. Therefore, the IPTCC should not be taken out of context, and it is not the sole condition or explanation for the crisis the world has been facing. The weather is not a silver bullet.

Nonetheless, incorporating meteorological components into the overall analysis could allow a better surveillance and prediction of the dynamic of the pandemic, as exemplified here by the evolution of the number of hospitalizations. As there is a delay between the IPTCC time series and hospitalizations, our index could help to forecast the pressure on the health system 2 to 3 weeks beforehand. This can be implemented and continuously updated very easily using the temperature and humidity forecasts provided by Météo-France stations. The evolution of the local or regional weather is a factor that could help the government to predict future epidemic waves and make decisions and communicate to protect population against SARS-CoV-2, or other respiratory diseases spread by airborne transmission.

## Supplementary Information

Supplementary Information.
