# Supplementary material for: Quantification of the effects of climatic conditions on French hospital admissions and deaths induced by SARS-CoV-2
Source: Sci Rep. 2021 Nov 8;11:21812. doi: 10.1038/s41598-021-01392-2 (PMC8575948; doi:10.1038/s41598-021-01392-2)
Supplement: Supplementary file 1 — Supplementary Information. [file 41598_2021_1392_MOESM1_ESM.pdf]

Supplementary Materials for

Quantification of the effects of climatic conditions on French hospital admissions and deaths induced by SARS-CoV-2

Hippolyte d'ALBIS - Dramane COULIBALY - Alix ROUMAGNAC - Eurico DE CARVALHO FILHO - Raphaël BERTRAND

This document includes:

1. Geometrical representations of the IPTCC index
2. Plots of the variables of interest for the 54 administrative regions
3. Correlation coefficients between variables
4. Dynamic responses following an increase in the 'false' IPTCC
5. Dynamic responses following an increase in the normalized temperature index
6. Granger causality in a model extended for air pollution indicators
7. Estimations of the epidemiological responses to a change in the IPTCC and in the air pollution indicators

1. <u>Geometrical representations of the IPTCC index</u>

We identified 5 intervals for the IPTCC that correspond to different suitability conditions for the spread of the virus. Following Roumagnac et al. (2021) a color code can be associated to each interval in Table A1. 2D and 3D representation of the IPCC as a function of relative humidity and temperature are then provided in Figures A1 and A2.

Table A1: Color code and intervals on the IPTCC value

IPTCC	Color code	Definition
[0,20[		Climatic conditions limiting airborne spread of the virus.
[20,75[		Climatic conditions becoming suitable to the airborne spread of the virus.
[75,90[		Climatic conditions suitable to the airborne spread of the virus.
[90,97[		Climatic conditions very suitable to the airborne spread of the virus.
[97,100]		Climatic conditions extremely suitable to the airborne spread of the virus.

Figure A1. IPTCC as a function of temperature and relative humidity, 2D representation

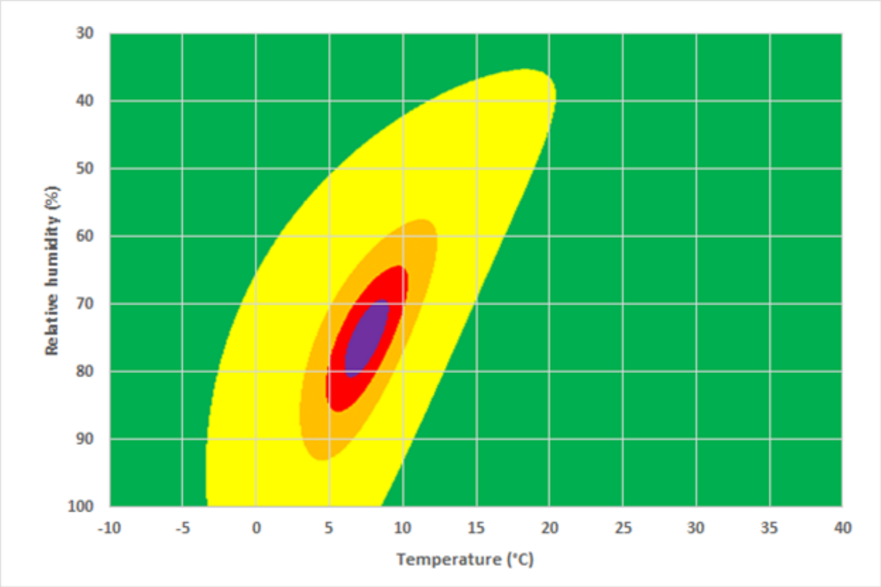

Figure A2. IPTCC as a function of temperature and relative humidity, 3D representation

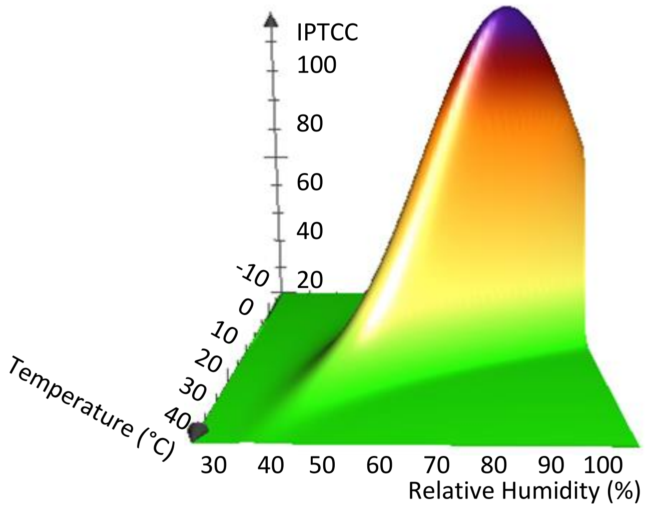

2. <u>Plots of the variables of interest for the 54 administrative regions</u>

Figure A3. Comparison of the time series of hospitalizations, deaths and IPTCC for each administrative region, from March 23, 2020 to January 10, 2021

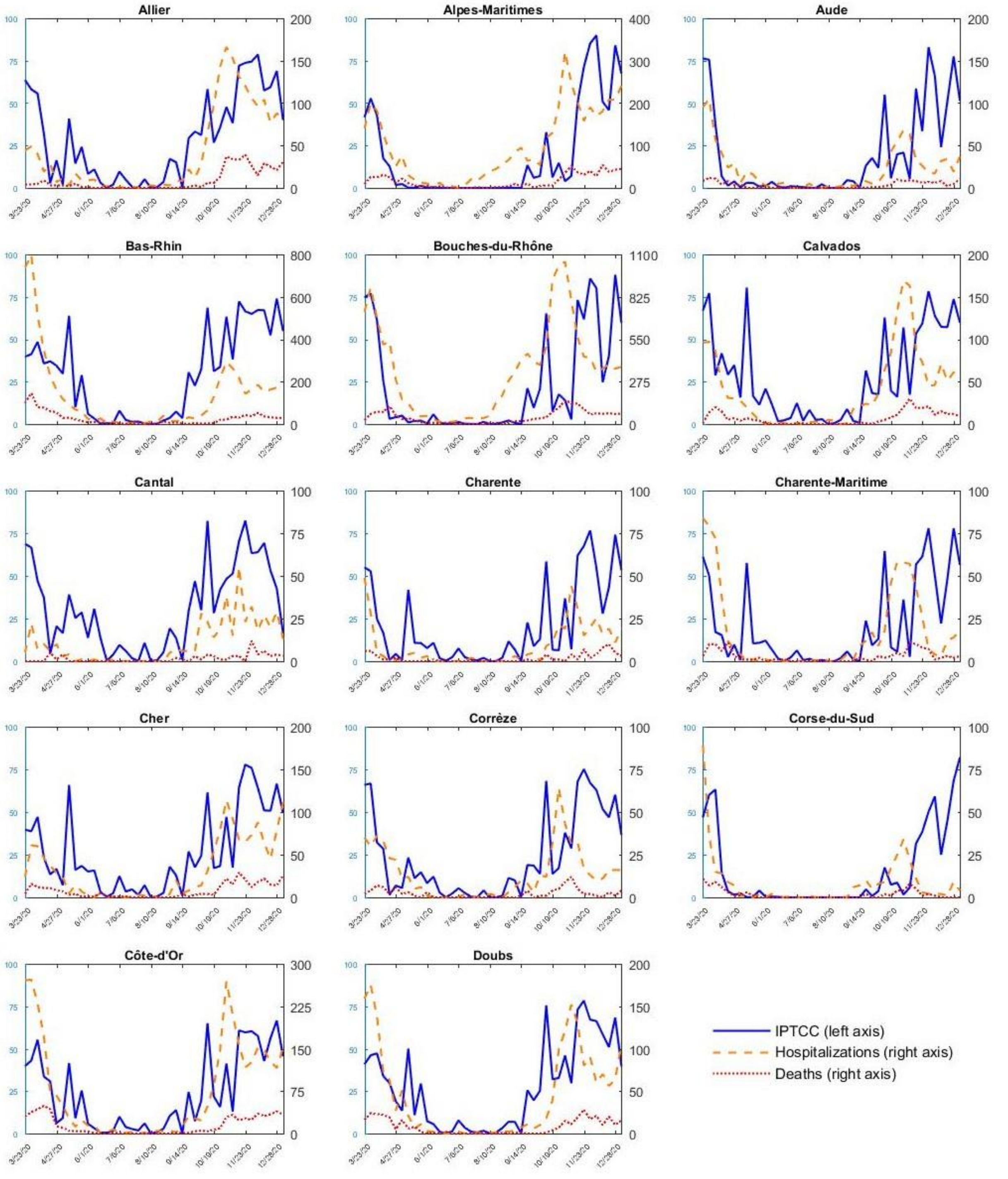

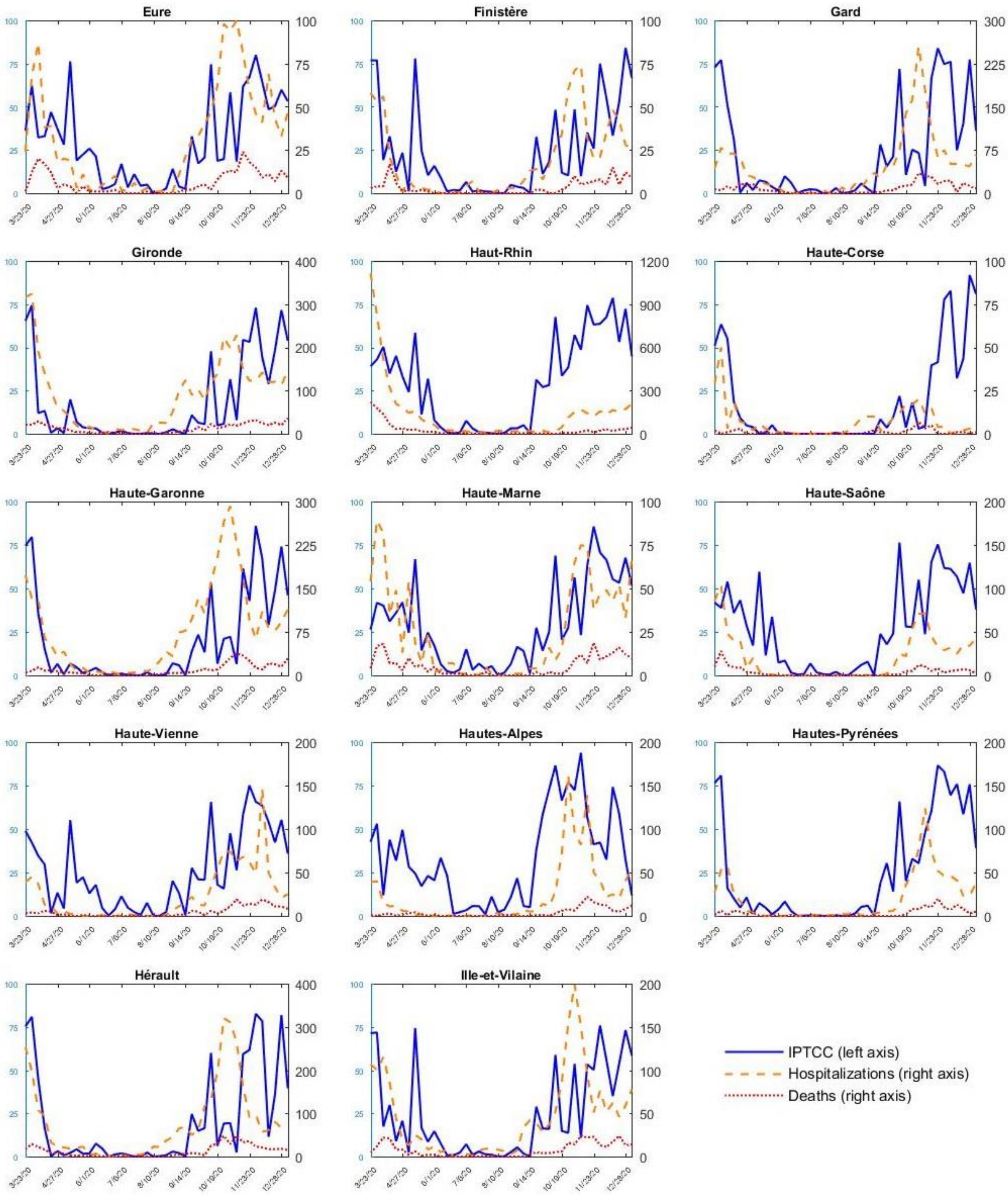
Eure
Finistère
Gard
Gironde
Haut-Rhin
Haute-Corse
Haute-Garonne
Haute-Marne
Haute-Saône
Haute-Vienne
Hautes-Alpes
Hautes-Pyrénées
Hérault
Ille-et-Vilaine
IPTCC (left axis)
Hospitalizations (right axis)
Deaths (right axis)

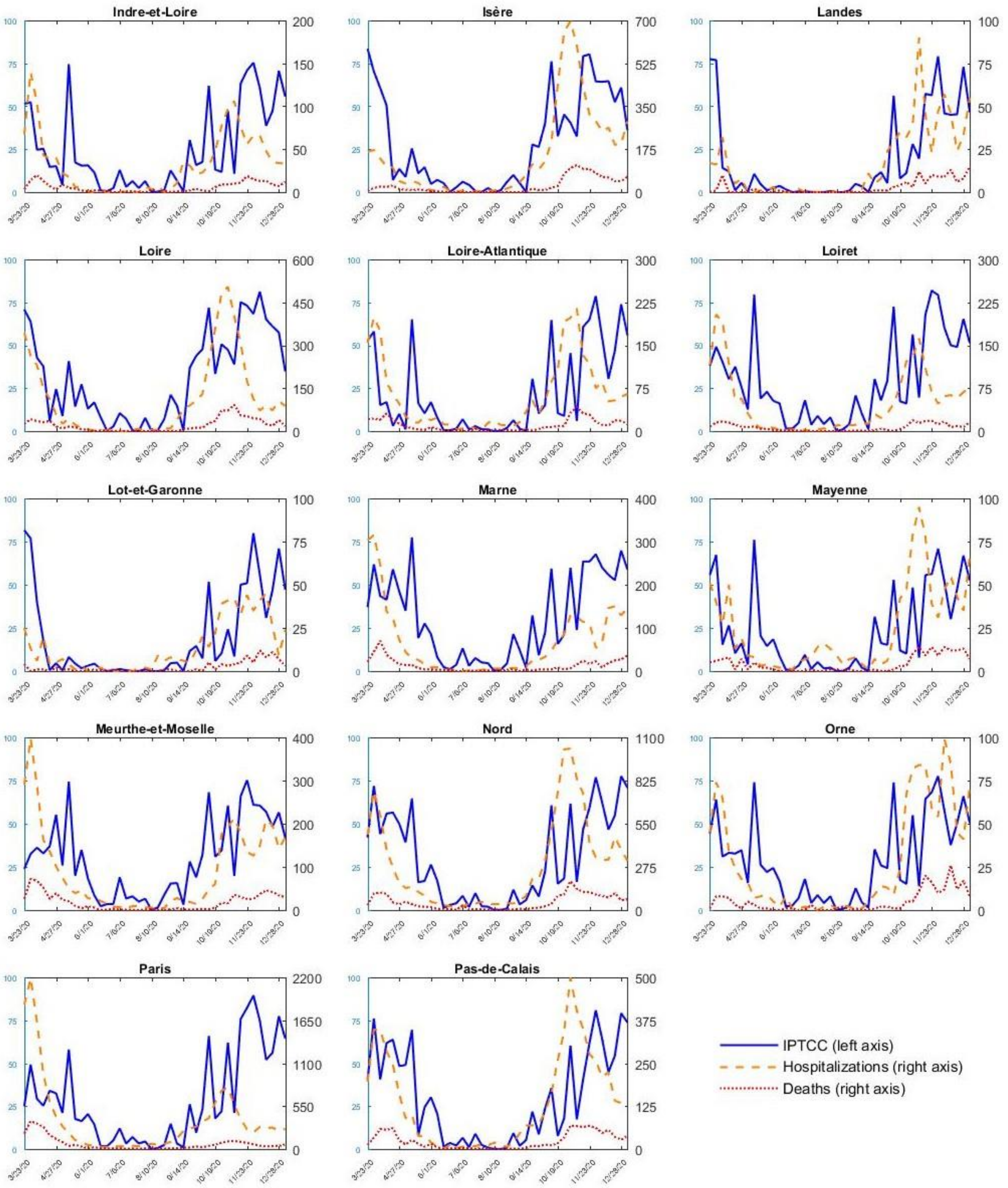
Indre-et-Loire
Isère
Landes
Loire
Loire-Atlantique
Loiret
Lot-et-Garonne
Marne
Mayenne
Meurthe-et-Moselle
Nord
Orne
Paris
Pas-de-Calais
IPTCC (left axis)
Hospitalizations (right axis)
Deaths (right axis)

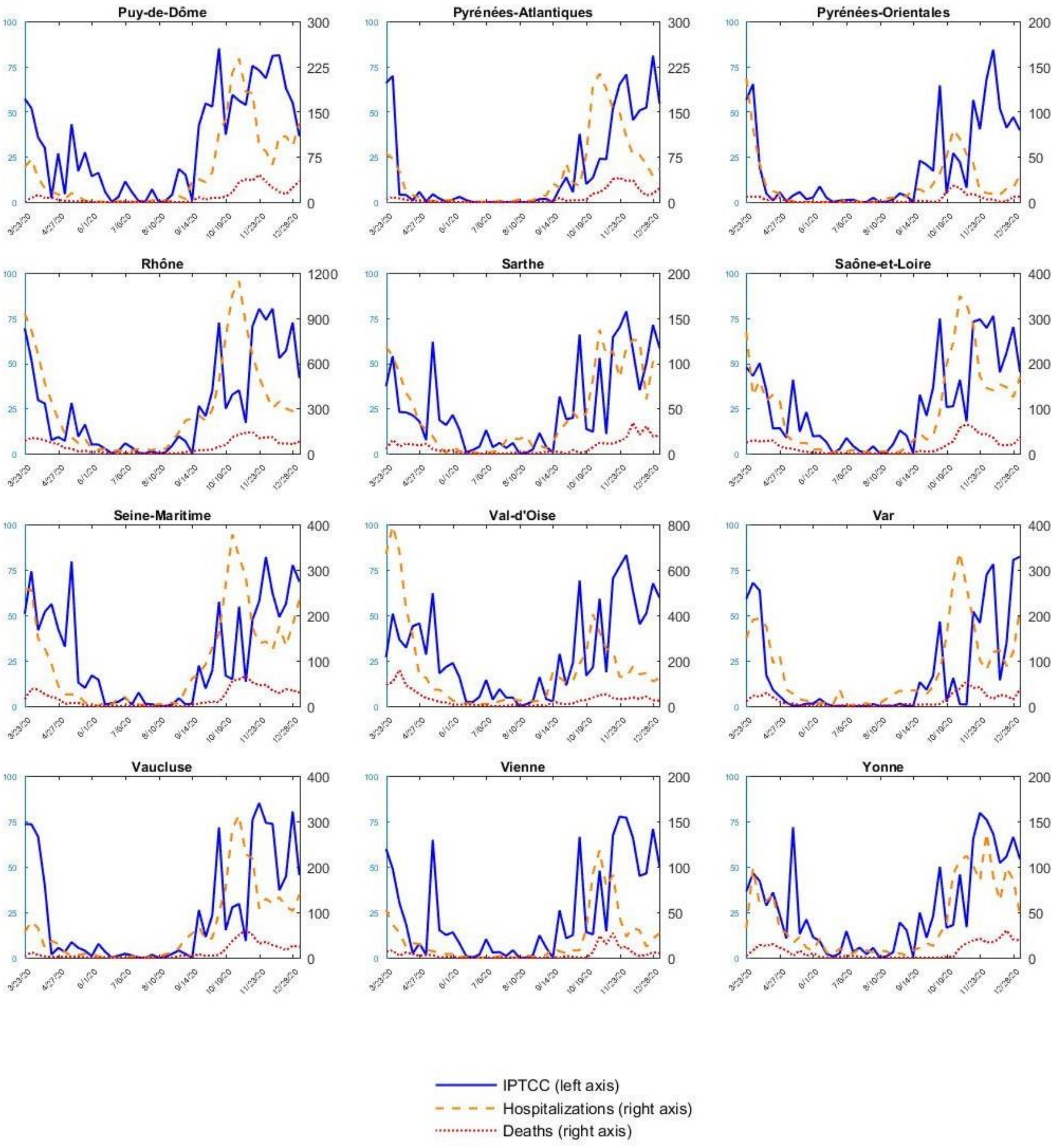
Puy-de-Dôme
Pyrénées-Atlantiques
Pyrénées-Orientales
Rhône
Sarthe
Saône-et-Loire
Seine-Maritime
Val-d'Oise
Var
Vaucluse
Vienne
Yonne
IPTCC (left axis)
Hospitalizations (right axis)
Deaths (right axis)

3. <u>Correlation coefficients between variables</u>

Table A2: Correlation coefficients between hospitalizations, deaths and the IPTCC

Region	corr(hospitlzation,death)	corr(hospitalization,IPTCC)	corr(death,IPTCC)
Allier	0.890	0.808	0.806
Alpes-Maritimes	0.800	0.588	0.663
Aude	0.838	0.573	0.640
Bas-Rhin	0.879	0.775	0.642
Bouches-du-Rhône	0.830	0.528	0.544
Calvados	0.817	0.675	0.713
Cantal	0.665	0.765	0.414
Charente	0.722	0.733	0.701
Charente-Maritime	0.771	0.445	0.459
Cher	0.897	0.686	0.701
Corrèze	0.812	0.598	0.412
Corse-du-Sud	0.750	0.382	0.327
Côte-d'Or	0.934	0.752	0.775
Doubs	0.894	0.802	0.732
Eure	0.902	0.651	0.686
Finistère	0.732	0.562	0.616
Gard	0.836	0.531	0.574
Gironde	0.818	0.577	0.665
Haut-Rhin	0.931	0.674	0.526
Haute-Corse	0.571	0.222	-0.001
Haute-Garonne	0.888	0.575	0.562
Haute-Marne	0.882	0.715	0.741
Haute-Saône	0.940	0.802	0.726
Haute-Vienne	0.895	0.707	0.736
Hautes-Alpes	0.756	0.745	0.551
Hautes-Pyrénées	0.822	0.755	0.697
Hérault	0.829	0.521	0.644
Ille-et-Vilaine	0.842	0.614	0.671
Indre-et-Loire	0.778	0.599	0.597
Isère	0.895	0.775	0.756
Landes	0.806	0.687	0.634
Loire	0.896	0.741	0.783
Loire-Atlantique	0.844	0.547	0.583
Loiret	0.806	0.610	0.671
Lot-et-Garonne	0.738	0.600	0.683
Marne	0.883	0.769	0.768
Mayenne	0.830	0.576	0.713
Meurthe-et-Moselle	0.910	0.703	0.670
Nord	0.885	0.699	0.784
Orne	0.822	0.676	0.643
Paris	0.904	0.493	0.550
Pas-de-Calais	0.890	0.710	0.805
Puy-de-Dôme	0.873	0.841	0.775
Pyrénées-Atlantiques	0.900	0.685	0.790
Pyrénées-Orientales	0.842	0.568	0.475
Rhône	0.895	0.682	0.732
Sarthe	0.795	0.645	0.674
Saône-et-Loire	0.959	0.740	0.754
Seine-Maritime	0.880	0.652	0.759
Val-d'Oise	0.859	0.580	0.639

Var	0.918	0.567	0.581
Vaucluse	0.903	0.660	0.683
Vienne	0.827	0.634	0.652
Yonne	0.815	0.737	0.695
National average	0.843	0.647	0.644

4. Dynamic responses following an increase in the 'false' IPTCC index

Figure A4: Responses of hospitalizations and deaths following an increase in the 'false' IPTCC index

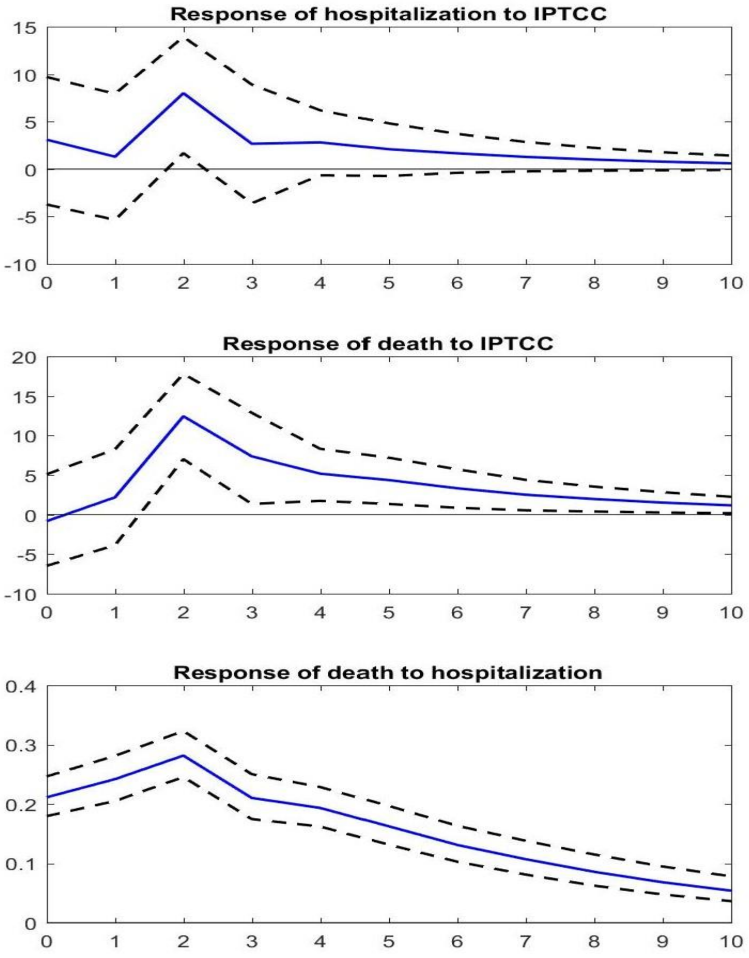

Notes: The solid line gives the estimated impulse responses. Dashed lines give the 90% confidence intervals generated by Monte Carlo with 5,000 repetitions. The size of the increase in the 'false' IPTCC is set to 10-point increase. The size of the increase in hospitalizations is set to one percent increase. The responses are the percent change in the number of hospitalizations and deaths.

5. <u>Dynamic responses following an increase in the normalized temperature index</u>

Figure A5: Responses of hospitalizations and deaths following an increase in the normalized temperature index.

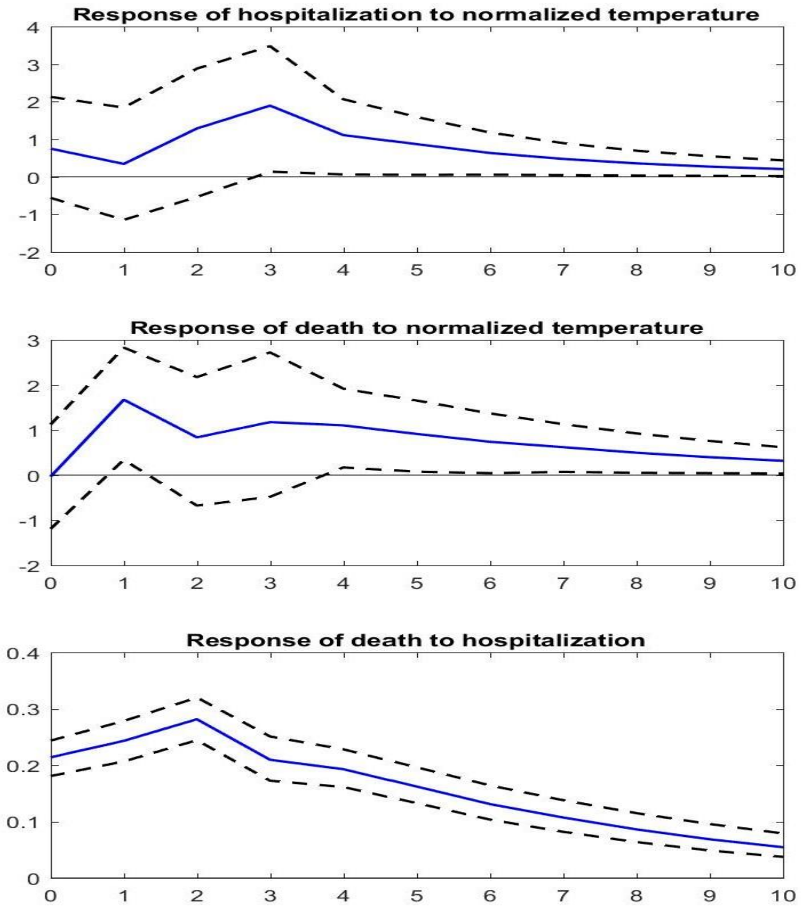

Notes: The solid line gives the estimated impulse responses. Dashed lines give the 90% confidence intervals generated by Monte Carlo with 5,000 repetitions. The size of the increase in the normalized temperature index is set to 10-point increase. The size of the increase in hospitalizations is set to one percent increase. The responses are the percent change in the number of hospitalizations and deaths.

6. <u>Granger causality in a model extended for air pollution indicators</u>

We have redone our computations of the Granger causality by including the following indicators: the concentration (in µg/m^3) of atmospheric particulate matter, either for particulates with a diameter of 10 µm (PM_{10}) and for those with a diameter of 2.5 µm ($PM_{2.5}$), of nitrogen dioxide (NO_2) and of ozone (O_3). We take the weekly average of the daily concentrations registered in all the Météo-France stations that monitor the pollution. Since the coverage of pollution is less important than that of climate, the number of regions used in the estimation is lower. It is equal to 21 for PM_{10}, 18 for $PM_{2.5}$ and NO_2, and 23 for O_3.

Equation (1) was replaced by:

$$y_{it} = \sum_{s=1}^{p} \alpha_s \, y_{it-s} + \sum_{s=1}^{p} \beta_s \, cl_{it-s} + \sum_{s=1}^{p} \gamma_s \, ap_{it-s} + \mu_i + \delta_i . t + \varepsilon_{it},$$

$$i = 1, \dots N \; and \; t = 1, \dots, T \qquad (A1)$$

where ap_{it-s} is the considered air pollution indicator. Remark that, given the small number of regions with data on air pollution a common national time effect is not included in this latter equation.

As previously, the null hypothesis of no Granger causality from climatic conditions to hospitalizations or deaths is H_0: $\beta_1 = \beta_2 = \dots = \beta_p$. The corresponding Wald test statistic is $W = \hat{\theta}' R' [\hat{\sigma}^2 R (X'X)^{-1} R']^{-1} R \hat{\theta}$ where $\hat{\theta}$ is the estimator of $\theta = (\alpha_1, \dots, \alpha_p, \beta_1, \dots, \beta_p, \gamma_1, \dots, \gamma_p)'$, R is a known $(p \times 2p)$ matrix with $R = [0: I_p]$, X is a $(N(T - p) \times 3p)$ matrix of $\tilde{y}_{it-1}, \dots, \tilde{y}_{it-p}, \widetilde{cl}_{it-1}, \dots, \widetilde{cl}_{it-p}, \widetilde{ap}_{it-1}, \dots, \widetilde{ap}_{it-p}$ that are the transformations of corresponding variables after removing region specific effects and region specific trends, and $\hat{\sigma}^2$ is the estimated variance of the residual in Equation (A1). Under the

null hypothesis, W follows a chi-squared distribution of a degree of freedom equal to p (which correspond to the number of constraints to be tested that corresponds to the lag length). In the same vein, based on Equations (A1), we also apply the Granger causality from air pollution to hospitalizations or deaths (controlling for climate conditions, IPTCC), with the corresponding hypothesis H_0: $\gamma_1 = \gamma_2 = \cdots = \gamma_p$.

Table A3 reports the results of Granger non-causality from IPTCC to hospitalizations and deaths controlling for and air pollution indicator and from air pollution to hospitalizations and deaths controlling for the IPTCC. When accounting for the potential impact of air pollution on hospitalizations and deaths, at the 5% level of significance (actually, even at the 1% level), we cannot accept the null hypothesis of no Granger causality from IPTCC to either hospitalizations or deaths induced by SARS-CoV-2. However, we cannot reject the null of hypothesis of no Granger causality from air pollution to either hospitalizations or deaths. This does not imply that air pollution does not contemporaneously affect hospitalizations and deaths, since Granger causality assesses how lag values of air pollution are useful to predict hospitalizations and deaths. The contemporaneous impact can be computed with the VAR by the response to an innovation or a structural shock of air pollution (see below).

Table A3: Granger causality from the IPTCC to hospitalizations and deaths, controlling for air pollution

Hypothesis	Test statistics	P-value
Model with measures of PM_{10}		
IPTCC does not Granger-cause hospitalizations	17.389	0.001
IPTCC does not Granger-cause deaths	28.478	0.000
Air pollution does not Granger-cause hospitalizations	2.775	0.428
Air pollution does not Granger-cause deaths	1.467	0.690
Model with measures of $PM_{2.5}$		
Hypothesis	Test statistics	P-value
IPTCC does not Granger-cause hospitalizations	12.830	0.005
IPTCC does not Granger-cause deaths	23.401	0.000
Air pollution does not Granger-cause hospitalizations	5.456	0.141
Air pollution does not Granger-cause deaths	4.869	0.182
Model with measures of NO_2		
Hypothesis	Test statistics	P-value
IPTCC does not Granger-cause hospitalizations	20.351	0.000
IPTCC does not Granger-cause deaths	28.828	0.000
Air pollution does not Granger-cause hospitalizations	1.513	0.679
Air pollution does not Granger-cause deaths	3.178	0.365
Model with measures of O_3		
Hypothesis	Test statistics	P-value
IPTCC does not Granger-cause hospitalizations	22.939	0.000
IPTCC does not Granger-cause deaths	33.400	0.000
Air pollution does not Granger-cause hospitalizations	2.093	0.553
Air pollution does not Granger-cause deaths	4.830	0.186

Notes: The test statistic is a Wald statistic which follows, under the null hypothesis, a chi-squared distribution of 3 (the number of constraints that corresponds to the lag length).

7. Estimations of the epidemiological responses to a change in the IPTCC in models that include an air pollution indicator

As for Granger causality, the estimations of the dynamic responses of the epidemiological variables to a change in the IPTCC can be done in a model augmented with an air pollution indicator (denoted as in A1, ap_{it} that can be the concentration of PM_{10}, $PM_{2.5}$, NO_2 or O_3). Equation (2) was replaced by:

$$Z_{it} = \sum_{s=1}^{p} A_s\, Z_{it-s} + \sum_{s=0}^{p} b_s\, cl_{it-s} + u_i + d_i.t + v_{it}, \;\; i = 1, \dots N \; and \; t = 1, \dots, T \qquad (A2)$$

where A_s are 3×3 matrices of coefficients associated with $Z_{it} = (ap_{it-s}, hosp_{it-s}, death_{it-s})'$, b_s are 3×1 vector of coefficient associated with cl_{it-s}, $u_i = (u_i^1, u_i^2, u_i^3)'$ is a vector of region fixed-effects; $d_i.t = (d_i^1, d_i^2, d_i^3)'.t$ represent region specific-time (linear) trend; $v_{it} = (v_{it}^1, v_{it}^2, v_{it}^3)'$ is a 3-dimensional vector of errors satisfying $E(v_{it}) = 0$ and $E(v_{it}v_{is}') = \Omega.\mathbb{1}\{t = s\}$ for all t and s. It is worth noting that air pollution is included in the endogenous vector Z_{it}, since it can be affected by the pandemic disease. As in Equation (A1), given the small number of regions with data on air variable a common national time effect is not included in Equation (A2).

As in Equation (2), to identify the response between endogenous variables (air pollution, deaths and hospitalization, we need to identify the structural shocks η_{it} of these endogenous variables as follow: $\eta_{it} = A_0 v_{it}$ where A_0 is (3×3) matrix such that $E(\eta_{it}\eta_{it}') = I_3$ or $A_0 A_0' = \Omega$. We identified A_0 based on Cholesky decomposition by setting A_0 as the unique lower-triangular Cholesky factor of Ω. This identification relies on the reasonable assumption that air pollution may contemporaneously influence hospitalizations and deaths, hospitalizations may contemporaneously influence deaths, while hospitalizations can

potentially influence air pollution only with, and deaths can potentially influence air pollution and hospitalizations only with lags. Results are reported in Figure A6. We first see that the control for air pollution does not qualitatively alter the dynamic response of hospitalizations and deaths to the IPTCC. Second, we see that the two measures of concentration of atmospheric particulate matter (namely PM_{10} and $PM_{2.5}$) have a significant impact on hospitalizations and deaths induced by SARS-CoV-2, while the concentrations of nitrogen dioxide (NO_2) and of ozone (O_3) have no impact. We remark that even if PM_{10} and $PM_{2.5}$ do not Granger cause hospitalizations and deaths, they have significant contemporaneous impact inducing a significant dynamic impact.

Figure A6. Dynamic responses of hospitalizations and deaths to IPTCC and air pollution

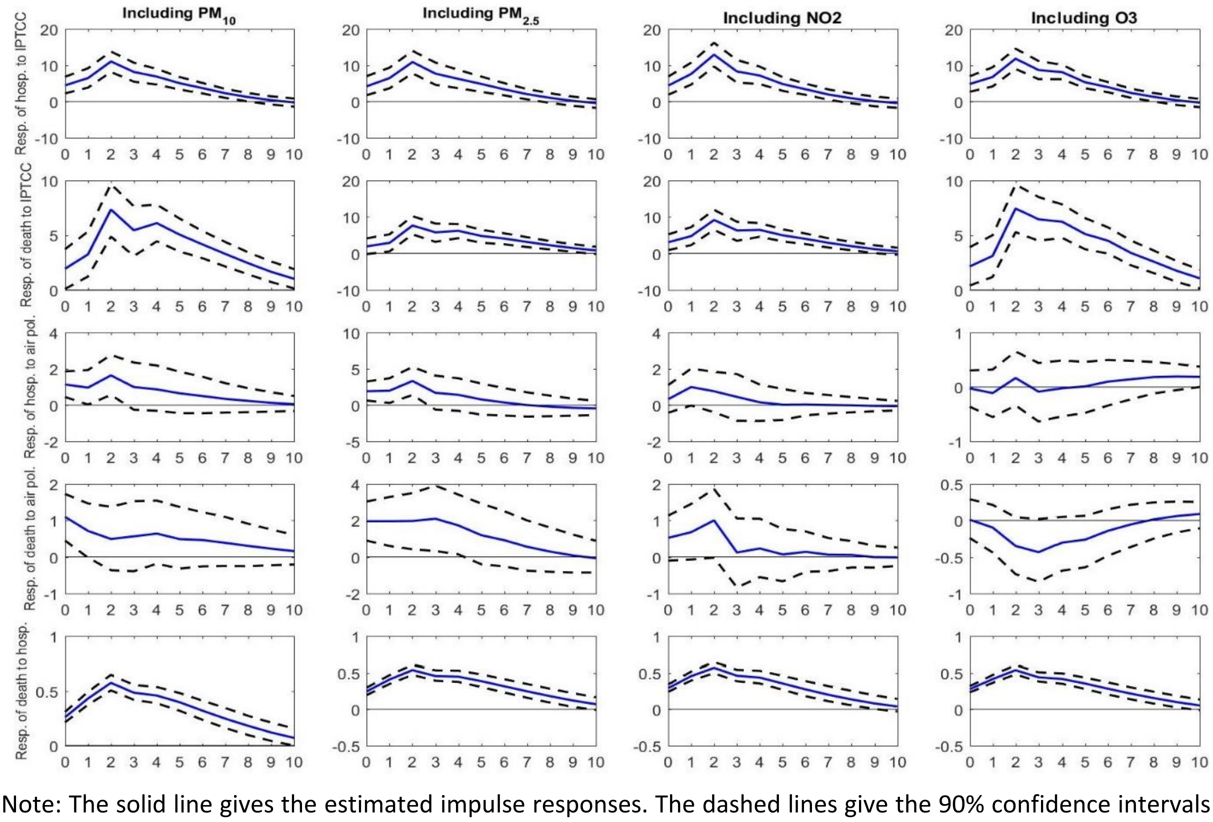

Note: The solid line gives the estimated impulse responses. The dashed lines give the 90% confidence intervals generated by Monte Carlo with 5,000 repetitions. The size of the increase in the IPTCC is set to a 10-point increase. The size of the increase in hospitalizations is set to a one-percent increase. The responses are the percentage change in the number of hospitalizations and deaths.
